# Maresin 1 Mitigates High Glucose-Induced Mouse Glomerular Mesangial Cell Injury by Inhibiting Inflammation and Fibrosis

**DOI:** 10.1155/2017/2438247

**Published:** 2017-01-15

**Authors:** Shi Tang, Chenlin Gao, Yang Long, Wei Huang, Jiao Chen, Fang Fan, Chunxia Jiang, Yong Xu

**Affiliations:** ^1^Endocrinology Department, The Affiliated Hospital of Southwest Medical University, Luzhou, Sichuan 646000, China; ^2^The Graduate School of Southwest Medical University, Luzhou, Sichuan 646000, China; ^3^State Key Laboratory of Quality Research in Chinese Medicine, Faculty of Chinese Medicine, Macau University of Science and Technology, Avenida Wai Long, Taipa, Macau

## Abstract

*Background*. Inflammation and fibrosis are the important pathophysiologic processes in diabetic nephropathy (DN). Maresin 1 is a potential anti-inflammatory lipid mediator, which has displayed powerful proresolving activities.* Aim*. We determine whether maresin 1 has protective effect on mouse glomerular mesangial cells (GMCs) induced by high glucose.* Methods*. We cultured GMCs stimulated by high glucose and categorized as follows: normal glucose group (5.6 mmol/L), high glucose group (30 mmol/L), mannitol group, maresin 1 intervention group (1, 10, and 100 nmol/L), maresin 1 and normal glucose group, and the N-acetylcysteine (NAC) intervention group (10 *μ*mol/L NAC). After 24 h, the expression of ROS, NLRP3, caspase-1, procaspase-1, IL-1*β*, and pro-IL-1*β* was detected by western-blot, RT-PCR, and immunofluorescence. After 48 h, the expression of TGF-*β*1 and FN was detected by RT-PCR and ELISA.* Results*. Compared with normal glucose group, the expression of ROS, NLRP3, caspase-1, IL-1*β*, TGF-*β*1, and FN increased in high glucose group (*P* < 0.05), but it decreased after the treatment of maresin 1 in different concentrations. On the contrary, the expression of procaspase-1 and pro-IL-1*β* protein was restrained by high glucose and enhanced by maresin 1 in a dose-dependent manner (*P* < 0.05).* Conclusion*. Maresin 1 can inhibit NLRP3 inflammasome, TGF-*β*1, and FN in GMCs; it may have protective effect on DN by mitigating the inflammation and early fibrosis.

## 1. Introduction

Diabetic nephropathy (DN) is unique and serious diabetic microvascular complications, which also is the leading cause of end-stage renal disease (ESRD) in the United States and other developed countries [1]. The primary pathological manifestations are glomerular mesangial lesions and extracellular matrix synthesis increase, which would lead to glomerular sclerosis and interstitial fibrosis ultimately [2]. The etiology and pathogenesis of DN are complex and have not been completely clarified. It has been well known that DN is a low-grade inflammatory disease. Metabolic disorder and hemodynamics changes caused by hyperglycemia persistently can trigger renal inflammation [3, 4]. Therefore, how to prevent DN effectively is the focus and difficulties in clinical work [5].

Reactive oxygen species (ROS) induced by sustained hyperglycemia in renal tissues may directly or indirectly activate nucleotide binding and oligomerization domain—like receptor family pyrin domain-containing 3 (NLRP3) inflammasome. NLRP3 inflammasome activates caspase-1 which can promote the maturation and secretion of inflammatory cytokine interleukin 1*β* (IL-1*β*) and trigger a powerful and endogenous inflammatory cascade reaction, which would cause the occurrence and development of DN [6, 7]. The activation of NLRP3 inflammasome includes two characteristics, the upregulation of NLRP3 and the production of caspase-1 and IL-1*β*. In macrophages, NF-КB can promote NLRP3 upregulation [8, 9]. The latest studies have shown that careful targeting of NLRP3 signaling pathways could be beneficial for the treatment of diabetes mellitus and diabetic nephropathy, so the NLRP3 inflammasome should be a new potential target for the treatment of diabetic nephropathy [10–12]. Additionally, researchers have found intraperitoneal injection stephania and piperine can reduce the expression of NLRP3 inflammasome, which would inhibit the progression of DN in diabetic rats [13].

Epithelial-mesenchymal transition (EMT) plays an important role in the early progression of renal fibrosis in DN [14]. Transforming growth factor-*β*1 (TGF-*β*1) which is highly expressed in DN renal tissue is a key regulator of EMT and is one of the important marks of renal fibrosis [15]. TGF-*β*1 can increase the expression of fibronectin (FN). Studies have shown that oxymatrine can prevent high glucose-induced renal EMT by inhibiting TGF-*β*1/Smad signaling pathway, thereby alleviating early DN renal fibrosis [16].

Numerous clinical studies have shown that n-3 polyunsaturated fatty acid can resist acute or chronic inflammation [17]. Maresin 1 is one of the latest families of anti-inflammatory lipid mediator which derives from n-3 polyunsaturated fatty acids and has displayed both anti-inflammatory and proresolving activities in zymosan-induced peritonitis [18]. Researches in inflammation-related disease have proved that maresin 1 plays a protective role in the body by limiting neutrophil infiltration, enhancing macrophage phagocytosis, decreasing the production of proinflammatory cytokines, inhibiting NF-КB activation, and so on [19, 20]. Recent studies show that maresin 1 not only had a significant role in terms of anti-inflammatory but also restrained the progression of pulmonary fibrosis by inhibiting EMT that is induced by TGF-*β*1 and by reducing the expression of FN [21]. However, the relationship between maresin 1 and pathogenesis of DN is unclear. In this study, we explored something new; we observed anti-inflammatory and antifibrosis effect of maresin 1 which is produced by inhibiting NLRP3 inflammasome activation and by lessening the TGF-*β*1-induced EMT on glomerular mesangial cells (GMCs) stimulated by high glucose.

## 2. Materials and Methods

### 2.1. Cell Culture and Stimulation

Mouse glomerular mesangial cells (GMCs) were purchased from Chinese Academy of Sciences and cultured in low glucose Dulbecco's Modified Eagle Medium (DMEM, Hyclone, USA) with 10% fetal bovine serum (FBS, BOVOGEN, Australia) at 37°C and 5% CO_2_. GMCs were used between the 2nd and 5th passages for all experiments. To observe the effect of maresin 1, we divided the cells into 8 groups: ① normal control group (NC group): 5.6 mmol/L glucose, ② osmotic pressure control group (OP group): 5.6 mmol/L glucose + 24.4 mmol/L mannitol, ③ high glucose group (HG group): 30 mmol/L glucose, ④ different concentrations of maresin 1 + high glucose group (M + HG group): the GMCs being treated with different concentration of maresin 1 (1, 10, and 100 nmol/L, Cayman Chemical, USA) for 30 min before being exposed to high glucose (30 mmol/L) [22, 23], 1 nmol/L maresin 1 + 30 mmol/L glucose (M1 + HG group), 10 nmol/L maresin 1 + 30 mmol/L glucose (M2 + HG group), 100 nmol/L maresin 1 + 30 mmol/L glucose (M3 + HG group), ⑤ maresin 1 + normal control group (M + NC group): the most obvious effect of maresin 1 concentration + 5.6 mmol/L glucose, ⑥ N-acetylcysteine (NAC) intervention group as positive control (10 *μ*mol/L NAC + HG group) [24, 25]. Each test was independently repeated more than 3 times.

### 2.2. Reverse-Transcription Polymerase Chain Reaction (RT-PCR)

Total RNA was isolated from GMCs using an RNA extraction kit (Beijing Tiangen Biotech, China). Total RNA was reverse transcribed with the reverse transcriptase kit (TOYOBO, Japan). All kits were used according to the manufacturer's instructions. RT-PCR was performed as previously described [26]. The annealing temperature of NLRP3, caspase-1, IL-1*β*, TGF-*β*1, and *β*-actin was 58.9°C, 60°C, 64.5°C, 59.8°C, and 54°C. The primer sequences (Shanghai biological Engineering Co. Ltd., China) were as follows: NLRP3 (Forward: 5′-AGA AGA GAC CAC GGC AGA AAG-3′, Reverse: 5′-CTT GGA ACC AGG TTG AGT GT-3′), caspase-1 (Forward: 5′-TGG AAG GTA GGC AAG ACT-3′, Reverse: 5′-ATA GTG GGC ATC TGG GTC-3′), IL-1*β* (Forward: 5′-GTC TTT CCC GTG ACC TTC-3′, Reverse: 5′-ATC TCG GAG CCT GTT AGT GC-3′), TGF-*β*1 (Forward: 5′-CTG TCC AAA CTA AGG CTC GC-3′, Reverse: 5′-AGA CAG CCA CTC AGG CGT AT-3′), and *β*-actin (Forward: 5′-ACC TCT ATG CCA ACA CAG TG-3′, Reverse: 5′-GGA CTC ATC GTA CTC CTG CT-3′).

### 2.3. Western Blotting

Total protein was extracted from GMCs using a protein extraction kit (Roche, USA). Protein concentrations were assessed by using BCA Protein Assay Kit (Bioworld Technology, USA). The OD value was measured at a wavelength of 562 nm, the standard curve (*r* > 0.99) calculated protein sample concentration. Western blotting was performed as previously described [27]. Immunoblotting was performed using NLRP3 antibody (rabbit, 1 : 800, CST, USA), caspase-1 antibody (rabbit, 1 : 1000, Abcam, USA), procaspase-1 antibody (rabbit, 1 : 1000, Abcam, USA), IL-1*β* (rabbit, 1 : 800, Abcam, USA), pro-IL-1*β* (rabbit, 1 : 1500, Abcam, USA), and GAPDH (rabbit, 1 : 10000, Bioworld Technology, USA). The second antibody of NLRP3, caspase-1, procaspase-1, IL-1*β*, and pro-IL-1*β* (1 : 2000, anti-rabbit) was obtained from the Beyotime Institute of Biotechnology, Shanghai, China. The proteins were detected with HRP chemiluminescence reagent (Millipore, USA) and images were captured with the UVP imaging system (Bio-Rad, USA).

### 2.4. ROS Assay

In order to detect the ROS, we used ROS detection kit (Nanjing Jiancheng Institute of Biotechnology, China). GMCs were grown in 6-well and 24-well plates and then were incubated with 10 *μ*mol/L DCFH-DA for 30 minutes at 37°C and 5% CO_2_. After washing with PBS for three times, 6-well cells were observed and imaged by fluorescence microscope. In addition, in order to obtain comparable data, 24-well cells were collected to detect the expression of ROS using ROS detection kit (Nanjing Jiancheng Institute of Biotechnology, China) by spectrophotometer with excitation wavelength at 488 nm and emission wavelength at 525 nm.

### 2.5. Enzyme-Linked Immunosorbent Assay (ELISA)

GMCs were grown in 24-well plates and were cultured with different medium at 37°C and 5% CO_2_. After 48 h, the levels of FN in supernatant of GMCs for each group were determined by using FN ELISA kits (Beijing Cheng Lin Biotechnology, China) according to manufacturer's instruction.

### 2.6. Immunofluorescence

GMCs were grown on coverslips in 6-well plates. After overnight adherence, cells were incubated with different compounds for 24 h as described above and then were fixed in 4% paraformaldehyde and then blocked with 5% rabbit serum. The cells were incubated overnight with the anti-NLRP3, caspase-1, and IL-1*β* primary antibodies (dilution 1 : 50) and incubated for 60 min with secondary antibody conjugated to the fluorescein isothiocyanate fluorescent dye (dilution 1 : 100). DAPI (4′,6′-diamino-2-phenylindole) was used to stain the nucleus in the cells. Images were taken with a laser scanning confocal microscope (Leica, Germany).

### 2.7. Statistical Analysis

All data are indicated as mean ± standard deviation (SD) and analyzed using one-way analysis of variance (ANOVA), followed by the LSD post hoc test for multiple comparisons (SPSS 20.0 statistical software). *P* < 0.05 was considered significant.

## 3. Results

### 3.1. Anti-Inflammatory Effect of Maresin 1 on GMCs Induced by High Glucose

#### 3.1.1. The Expression of NLRP3, Caspase-1, Procaspase-1, IL-1*β*, and Pro-IL-1*β* in GMCs Induced by 30 mmol/L Glucose for Each Group

**Figure 1:**
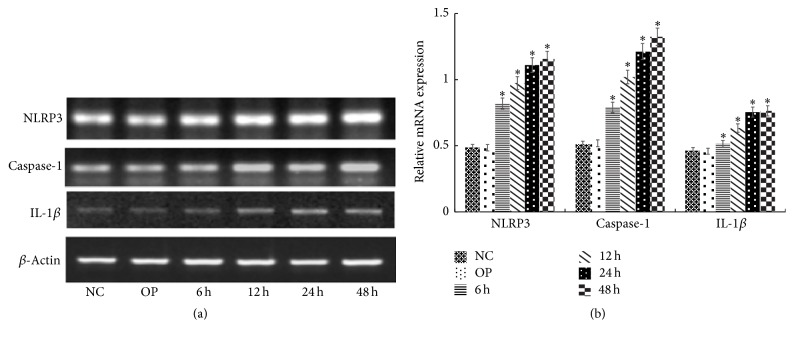
The mRNA expression of NLRP3, caspase-1, and IL-1*β* in mesangial cells treated with 30 mmol/L glucose for different time. (a) RT-PCR strip chart for different time. NLRP3, caspase-1, and IL-1*β* mRNA increased significantly after 6 h, 12 h, 24 h, and 48 h of exposure to 30 mmol/L glucose. (b) The corresponding relative gray value statistics graph of the mRNA level. ^*∗*^*P* < 0.05 versus NC group.

**Figure 2:**
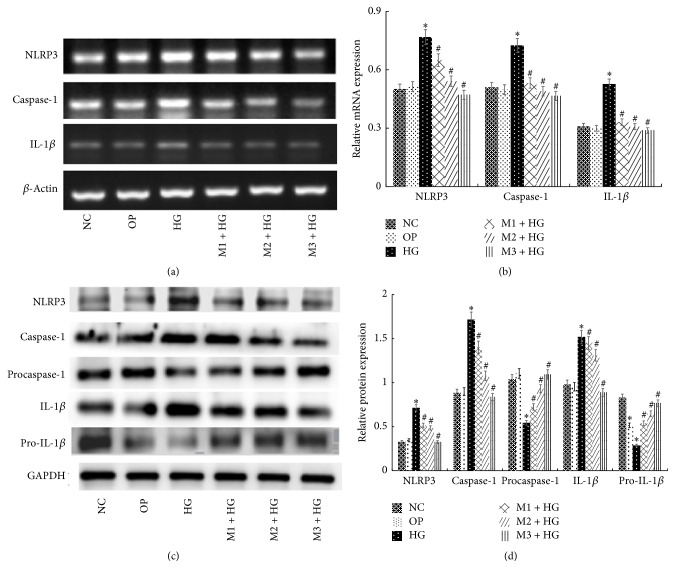
The mRNA and protein level of NLRP3, caspase-1, procaspase-1, IL-1*β*, and pro-IL-1*β* in each group. (a) RT-PCR detected the expression of NLRP3, caspase-1, and IL-1*β* mRNA after intervened by high glucose and maresin 1 for 24 h in GMCs, and the mRNA expression of NLRP3, caspase-1, and IL-1*β* increased in HG group significantly, but compared with HG group, it decreased obviously in M + HG group as a concentration-dependent manner of maresin 1. (b) The corresponding relative gray value statistics graph of the mRNA level. ^*∗*^*P* < 0.05 versus NC group, ^#^*P* < 0.05 versus HG group. (c) Western-blot detected the expression NLRP3, caspase-1, procaspase-1, IL-1*β*, and pro-IL-1*β* protein after intervened by high glucose and maresin 1 for 24 h in GMCs, and the protein expression of NLRP3, caspase-1, and IL-1*β* increased and procaspase-1 and pro-IL-1*β* decreased in HG group significantly, but compared with HG group, the protein expression of NLRP3, caspase-1, and IL-1*β* decreased and procaspase-1 and pro-IL-1*β* increased obviously in M + HG group as a concentration-dependent manner of maresin 1. (d) The corresponding relative gray value statistics graph of the protein level. ^*∗*^*P* < 0.05 versus NC group, ^#^*P* < 0.05 versus HG group.

**Figure 3:**
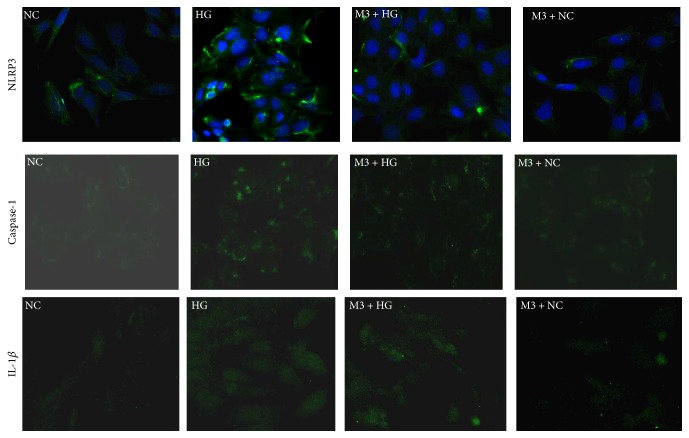
The level of NLRP3, caspase-1, and IL-1*β* in each group detected by immunofluorescence. The expression of NLRP3, caspase-1, and IL-1*β* increased in HG group significantly, but it decreased obviously in M3 + HG group, and there was no difference between NC group and NC + M3 group.

**Figure 4:**
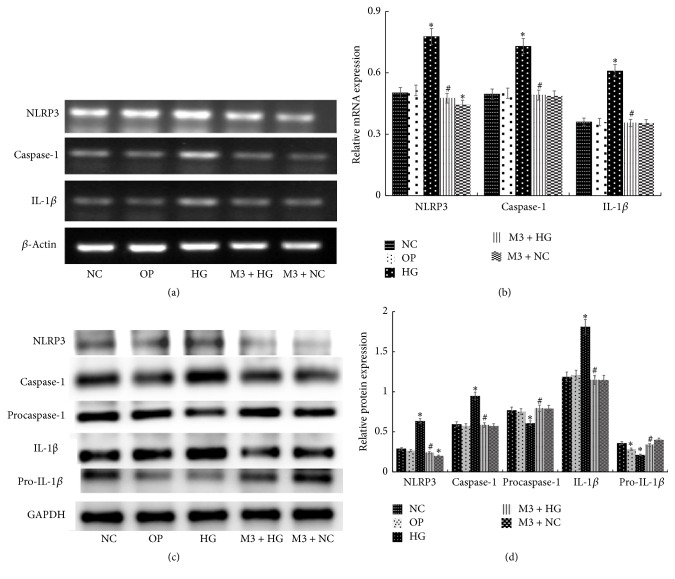
The level of NLRP3, caspase-1, P-caspase-1, IL-1*β*, and P-IL-1*β* in each group. (a) RT-PCR detected the expression NLRP3, caspase-1, and IL-1*β* mRNA after intervened by high glucose and 100 nmol/L maresin 1 in GMCs, and in HG group, the mRNA expression of NLRP3, caspase-1, and IL-1*β* was higher than that in NC group, but for M3 + HG group, the expression reduced visibly compared to the HG group. (b) The corresponding relative gray value statistics graph of the mRNA level. ^*∗*^*P* < 0.05 versus NC group, ^#^*P* < 0.05 versus HG group. (c) Western-blot detected the expression of NLRP3, caspase-1, P-caspase-1, IL-1*β*, and P-IL-1*β* protein after intervened by high glucose and 100 nmol/L maresin 1 in GMCs, and the protein expression of NLRP3, caspase-1, and IL-1*β* was higher than that in NC group, but for M3 + HG group, the expression reduced visibly compared to the HG group. The expression of P-caspase-1 and P-IL-1*β* was just the reverse. (d) The corresponding relative gray value statistics graph of the protein level. ^*∗*^*P* < 0.05 versus NC group, ^#^*P* < 0.05 versus HG group.

Compared with NC group, the mRNA expression of NLRP3, caspase-1, and IL-1*β* increased significantly after exposing to 30 mmol/L glucose for 6 h, 12 h, 24 h, and 48 h (*P* < 0.05), and the expression was positively correlated with time changes (Figures 1(a) and 1(b)). Moreover, in M + HG group for 24 h, the protein and mRNA expression of NLRP3, caspase-1, and IL-1*β* decreased, but procaspase-1 and pro-IL-1*β* were enhanced obviously as a concentration-dependent manner of maresin 1 compared with HG group (*P* < 0.05) (Figures 2(a), 2(b), 2(c), and 2(d)). And we used immunofluorescence to detect the level of NLRP3, caspase-1, and IL-1*β* in each group, and we observed the same change (Figure 3). There were no apparent differences among NC group, OP group, and M + NC group (*P* > 0.05) (Figures 4(a), 4(b), 4(c), and 4(d)).

#### 3.1.2. The ROS Level of GMCs Which Were Treated with NAC and Different Doses of Maresin 1 for 30 min and Were Exposed to High Glucose (30 mmol/L) for 24 h by Using Spectrophotometer and Fluorescence Microscope

**Figure 5:**
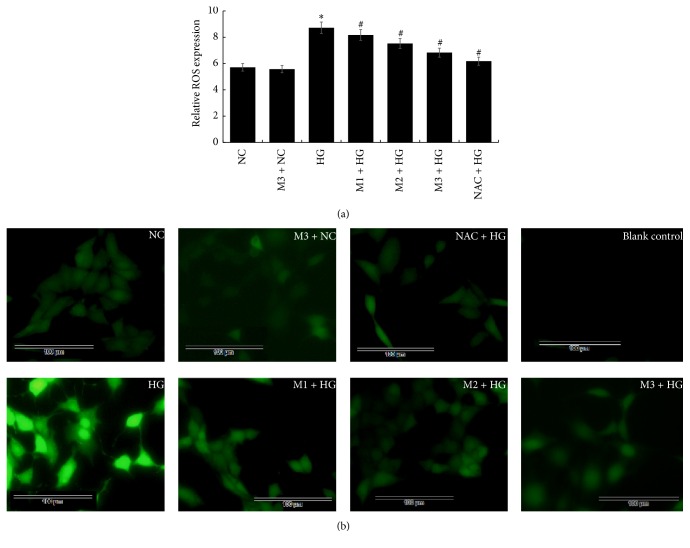
(a) DCFH-DA detected the expression of ROS in GMC after the treatment of NAC and maresin 1; ^*∗*^*P* < 0.05 versus NC group, ^#^*P* < 0.05 versus HG group. (b) GMCs were stained with DCFH-DA probe and images were captured using fluorescence microscope. Compared with NC group, the ROS generation increased in HG group, but it decreased in NAC + HG and M + HG group in a concentration-dependent manner.

As ROS plays an important role in high glucose-induced damage, we explored ROS level in GMCs using DCFH-DA. In spectrophotometer analysis, we observed that high glucose caused a significant ROS generation in GMCs (*P* < 0.05) compared with NC group. Notably, ROS generation was significantly inhibited by NAC and maresin 1 (1, 10, and 100 nmol/L) (*P* < 0.05), and the ROS level decreased in a concentration-dependent manner of maresin 1 (Figure 5(a)). Meanwhile, we got a similar result by using fluorescence microscopy (Figure 5(b)).

### 3.2. Antifibrosis Effect of Maresin 1 on GMCs Induced by High Glucose

**Figure 6:**
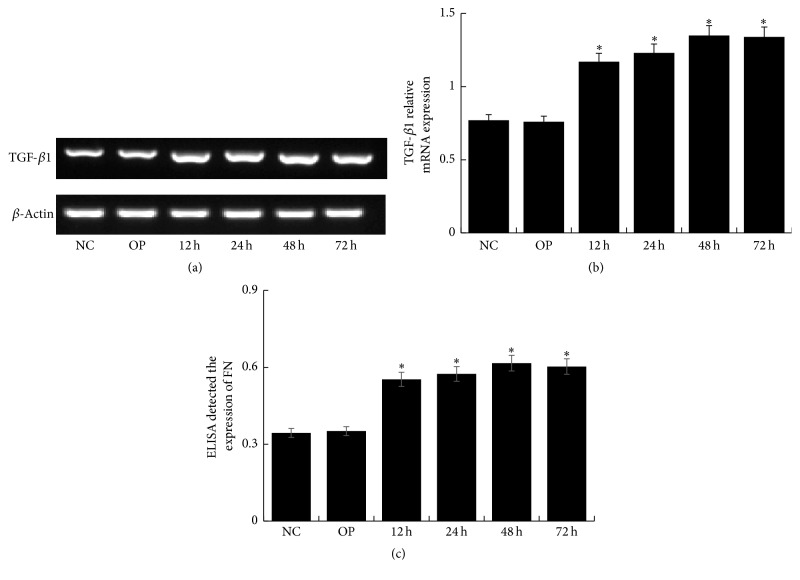
The expression of TGF-*β*1 and FN in mesangial cells treated with 30 mmol/L glucose for different time. (a) RT-PCR strip chart for different time. Compared with NC group, the expression of TGF-*β*1 upregulated in HG group for different time, especially for 48 h. (b) The corresponding relative gray value statistics graph of the mRNA level. ^*∗*^*P* < 0.05 versus NC group. (c) FN ELISA kit detected the level of FN in supernatant of GMCs treated with 30 mmol/L glucose for different time. Compared with NC group, the expression of FN upregulated in HG group for different time, especially for 48 h; ^*∗*^*P* < 0.05 versus NC group.

**Figure 7:**
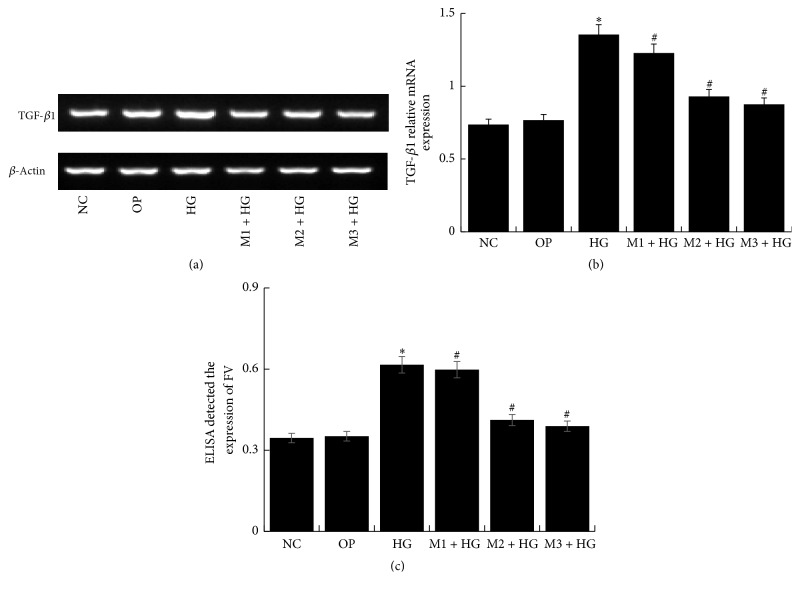
The expression of TGF-*β*1 and FN in each group. (a) Expression of TGF-*β*1 in GMCs which were treated with different does of maresin 1 for 30 min and were exposed to high glucose (30 mmol/L) for 48 h. Compared with NC group, expression of TGF-*β*1 was significantly increased in HG group, and compared with HG group, expression of TGF-*β*1 was significantly lower in M + HG group, and there were effective changes in M2 + HG group. (b) The corresponding relative gray value statistics graph of the mRNA level. ^*∗*^*P* < 0.05 versus NC group, ^#^*P* < 0.05 versus HG group. (c) FN ELISA kit detected the level of FN in each supernatant of GMCs; we detected the similar change with TGF-*β*1; ^*∗*^*P* < 0.05 versus NC group, ^#^*P* < 0.05 versus HG group.

**Figure 8:**
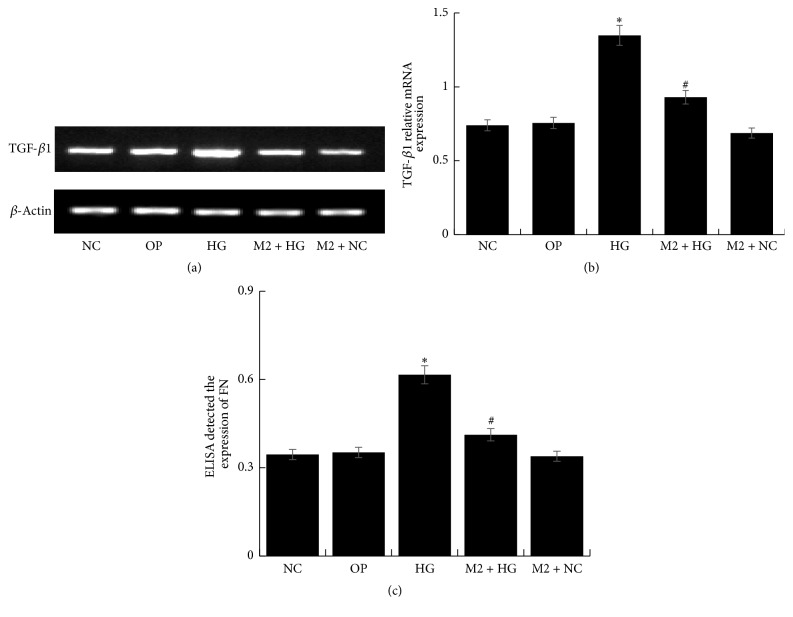
The level of TGF-*β*1 and FN in each group. (a) RT-PCR detected the expression of TGF-*β*1 mRNA after the treatment of maresin 1 for 48 h; compared with HG group, TGF-*β*1 mRNA was significantly decreased in M2 + HG group. (b) The corresponding relative gray value statistics graph of the mRNA level. ^*∗*^*P* < 0.05 versus NC group, ^#^*P* < 0.05 versus HG group. (c) FN ELISA kit detected the level of FN in supernatant of GMCs after the treatment of maresin 1; ^*∗*^*P* < 0.05 versus NC group, ^#^*P* < 0.05 versus HG group.

Compared with NC group, the expression of TGF-*β*1 and FN upregulated in HG group for different time (*P* < 0.05), especially for 48 h (Figures 6(a), 6(b), and 6(c)). However, it downregulated significantly in M + HG group by a negative correlation with concentration of maresin 1 (*P* < 0.05) (Figures 7(a), 7(b), and 7(c)). It seemed that 10 nmol/L maresin 1 was effective enough for counteracting the expression of TGF-*β*1 and FN enhanced by high glucose. Therefore, we chose 10 nmol/L as a protective concentration in the following experiments. And then, we detected that the mRNA level of TGF-*β*1 and the expression of FN decreased significantly in M2 + HG group compared with HG group (*P* < 0.05), and there was no significant difference between NC group and other groups (Figures 8(a), 8(b), and 8(c)).

## 4. Discussion

Diabetic nephropathy (DN) is the common and serious microvascular complications of diabetes; it can be divided into early stage kidney disease and clinical kidney disease according to the course of the disease. Once in clinical kidney disease, it will be irreversible, and there is little effective treatment for DN. Finally, the patient who was tortured by DN can be kept alive only by blood purification treatment. In western country, more than 50% of all patients who need blood purification treatment because of renal failure were caused by DN. Therefore, how to effectively delay or prevent the development of DN is a hot and difficult topic that the researchers explored now, but the mechanism of DN is unclear. Recent studies suggested that the chronic low-level inflammation mediated by natural immune plays a central role in the occurrence and development of DN [4]. The increasing secretion of cytokine and proinflammatory media would activate inflammatory response in local, accompanied with mesangial extracellular matrix accumulation, glomerular sclerosis, and tubular interstitial fibrosis [3]. It shows that inflammatory reaction would lead to injury by the pathological process of collagen deposition and fibrosis [3]. And the persistent inflammation and fibrosis in renal tissues are an important pathophysiological basis for DN in chronic hyperglycemia condition. So if we can resist this metabolic inflammation and early renal fibrosis, the renal damage process may be delayed significantly [13, 28].

Increasing evidence supported that proresolving mediators, such as LXs, resolvins, and protectins, can attenuate diabetes-related pathologies, including kidney disease and adipose inflammation [29]. Maresin 1 (7R, 14S-dihydroxy-docosa-4Z, 8E, 10E, 12Z, 16Z, 19Z-hexaenoic acids) produced by macrophages from docosahexaenoic acid (DHA) exerts potent proresolving and tissue homeostatic actions [30]. It is synthesised by lipoxygenase enzyme oxidation pathway in inflammation subsidising period and conjugates triene double bond [31]. Studies have confirmed that maresin 1 can suppress neutrophil infiltration and adhesion and reduce proinflammatory mediators such as TNF-*α*, IL-1*β*, and IL-6 in mice induced by acute lung injury [32]. Gong et al. have confirmed that maresin 1 and resolvin D2 can successfully prevent atheroprogression, suggesting that resolving lipid mediators potentially represent an innovative strategy to resolve arterial inflammation [33]. Meanwhile, maresin 1 also inhibits TGF-*β*1-induced EMT in alveolar type II epithelial cells by restoring epithelial marker (E-Cadherin) and inhibiting fibroblast phenotypes (fibronectin and a-SMA), which alleviate progress of lung fibrosis [21]. Additionally, maresin 1 has antioxidative and anti-inflammatory properties in CCl4 induced liver injury whose possible mechanisms are partly implicated in its abilities to inhibit ROS generation, activation of NF-КB, and MAPK pathway [34]. Furthermore, Hong et al. found maresin-like mediators (14,22-dihydroxy-docosa-4Z, 7Z, 10Z, 12E, 16Z, 19Z-hexaenoic acids) were produced by leukocytes and PLT and involved in wound healing by restoring reparative functions to diabetic macrophages (Mfs); additionally, it could ameliorate Mfs inflammatory activation and had the potential to suppress the chronic inflammation in diabetic wounds caused by activation of Mfs [35].

Inflammation is an innate immune response which is considered a common basis for diabetes. Inflammasomes are multiprotein platforms which act as cytoplasmatic sensors for stimulation with Pathogen Associated Molecular Pattern (PAMPs) and Damage-Associated Molecular Patterns (DAMPs) leading to an infectious/pathogenic or sterile inflammation [36]. So far, different inflammasomes have been identified, including nucleotide-binding oligomerization domain-like receptors (NLRs) and absent in melanoma 2 (AIM2) [37]. NLRP3 inflammasome is the most distinctive member in NLR family, which comprises NLRP3, apoptosis associated speck like protein (ASC), and the serine protease caspase-1 [37]. In metabolic diseases, the expression of NLRP3 inflammasome will increase, and now more and more evidence suggests that NLRP3 inflammasome plays an important role in many noninfectious inflammatory diseases, such as gout, atherosclerosis, and diabetes [38]. The mechanism of NLRP3 activation and IL-1*β* secretion is not clear. The secretion of IL-1*β* needs to activate caspase-1, and dead cell releases endogenous DAMPs, activating caspase-1 to promote the secretion of IL-1*β* by NLRP3 [33]. Researches show that in this process reactive oxygen species (ROS) formation was also thought to be related to the oligomerization reaction of NLRP3, and the endoplasmic reticulum was one of the important sources of ROS. ROS can cause NLRP3 activation by resolving the interaction of protein (TXNIP) from thioredoxin [39]. Previous studies have found that sustained hyperglycemia triggers the production of ROS, which directly or by stimulating the release of thioredoxin interacting protein (TXNIP) indirectly activate NLRP3 inflammasome formation. Then procaspase-1 will form an active caspase-1 p10/20 tetramer by self-splicing easily. The activated caspase-1 can process pro-IL-1*β* hydrolyze into its mature form IL-1*β* and induce inflammation cascade effect, which could mediated the occurrence of DN [40, 41]. Our research demonstrated that high glucose can increase mRNA expression of NLRP3, caspase-1, and IL-1*β* in GMCs and can cause a significant ROS generation in GMCs (*P* < 0.05) compared with NC group. These suggest that ROS-NLRP3-IL-1*β* plays an important role in high glucose-induced damage in GMCs. Besides, our results firstly detected that maresin 1 can inhibit the ROS generation and the activation of NLRP3 inflammasome pathway that is induced by high glucose in GMCs in a dose-dependent manner. These provided an important molecular and cellular foundation for elucidating the inflammatory response mechanisms of DN and suggested a new target and strategy for the prevention and treatment of DN.

TGF-*β*1 is a key regulator of EMT in pathological processes of glomerular sclerosis in DN, which participate in the renal fibrosis of DN commonly [14, 15]. Extracellular high glucose can be used as profibrotic factors in GMCs to promote the synthesis and secretion of TGF-*β*1 and increase the expression of FN that would prompt deposition of extracellular matrix and destruct the complete basement membrane. Finally, EMT and stable mesenchymal phenotype occurred [42]. This study in vitro found that high glucose could upregulate the expression of TGF-*β*1 and FN in GMCs in time dependence, suggesting that the high glucose may promote glomerular sclerosis and renal fibrosis in DN. What is more, maresin 1 could restrain the expression of TGF-*β*1 and FN induced by high glucose in GMCs in a dose-dependent manner, indicating that maresin 1 may inhibit EMT in GMCs and reduce DN glomerular fibrosis. Therefore, we hypothesized that maresin 1 potentially offers a therapeutic option for better treatment of DN through its anti-inflammatory effect and inhibition of renal fibrosis. However, this experiment is not enough; since the limit experimental time, we failed to investigate the specific mechanism of action, which needs the further basis experiments to prove.

In conclusion, maresin 1 can reduce the expression of NLRP3, caspase-1, IL-1*β*, and ROS generation that is induced by high glucose in GMCs; furthermore, the increased expression of TGF-*β*1 and FN following with high glucose in GMCs can also be reversed. And the mechanism may be related to the inhibition of NLRP3 inflammasome and early renal fibrosis growth factor TGF-*β*1 and FN induced by EMT.

So, maresin 1 may have protective effect on diabetic nephropathy by mitigating the inflammation and early fibrosis.

## References

[1] Donath M. Y. (2014). Targeting inflammation in the treatment of type 2 diabetes: time to start. *Nature Reviews Drug Discovery*.

[2] Kanwar Y. S., Sun L., Xie P., Liu F., Chen S. (2011). A glimpse of various pathogenetic mechanisms of diabetic nephropathy. *Annual Review of Pathology: Mechanisms of Disease*.

[3] Wada J., Makino H. (2013). Inflammation and the pathogenesis of diabetic nephropathy. *Clinical Science*.

[4] García-García P. M., Getino-Melián M. A., Domínguez-Pimentel V., Navarro-González J. F. (2014). Inflammation in diabetic kidney disease. *World Journal of Diabetes*.

[5] Tang S. C. W., Chan G. C. W., Lai K. N. (2016). Recent advances in managing and understanding diabetic nephropathy. *F1000Research*.

[6] De Nardo D., Latz E. (2011). NLRP3 inflammasomes link inflammation and metabolic disease. *Trends in Immunology*.

[7] Wang C., Pan Y., Zhang Q.-Y., Wang F.-M., Kong L.-D. (2012). Quercetin and allopurinol ameliorate kidney injury in STZ-treated rats with regulation of renal NLRP3 inflammasome activation and lipid accumulation. *PLoS ONE*.

[8] Bryant C., Fitzgerald K. A. (2009). Molecular mechanisms involved in inflammasome activation. *Trends in Cell Biology*.

[9] Bauernfeind F. G., Horvath G., Stutz A. (2009). Cutting edge: NF-*κ*B activating pattern recognition and cytokine receptors license NLRP3 inflammasome activation by regulating NLRP3 expression. *Journal of Immunology*.

[10] Lee H.-M., Kim J.-J., Kim H. J., Shong M., Ku B. J., Jo E.-K. (2013). Upregulated NLRP3 inflammasome activation in patients with type 2 diabetes. *Diabetes*.

[11] Wada J., Makino H. (2016). Innate immunity in diabetes and diabetic nephropathy. *Nature Reviews Nephrology*.

[12] Qiu Y., Tang L. (2016). Roles of the NLRP3 inflammasome in the pathogenesis of diabetic nephropathy. *Pharmacological Research*.

[13] Samra Y. A., Said H. S., Elsherbiny N. M., Liou G. I., El-Shishtawy M. M., Eissa L. A. (2016). Cepharanthine and Piperine ameliorate diabetic nephropathy in rats: role of NF-*κ*B and NLRP3 inflammasome. *Life Sciences*.

[14] Li Y., Kang Y. S., Dai C., Kiss L. P., Wen X., Liu Y. (2008). Epithelial-to-mesenchymal transition is a potential pathway leading to podocyte dysfunction and proteinuria. *The American Journal of Pathology*.

[15] Lan H. Y. (2011). Diverse roles of TGF-*β*/Smads in renal fibrosis and inflammation. *International Journal of Biological Sciences*.

[16] Liu L., Wang Y., Yan R. (2016). Oxymatrine inhibits renal tubular EMT induced by high glucose via upregulation of SnoN and inhibition of TGF-*β*1/Smad signaling pathway. *PLOS ONE*.

[17] Simopoulos A. P. (2002). Omega-3 fatty acids in inflammation and autoimmune diseases. *Journal of the American College of Nutrition*.

[18] Serhan C. N. (2014). Pro-resolving lipid mediators are leads for resolution physiology. *Nature*.

[19] Dalli J., Zhu M., Vlasenko N. A. (2013). The novel 13S,14S-epoxy-maresin is converted by human macrophages to maresin 1 (MaR1), inhibits leukotriene A_4_ hydrolase (LTA_4_H), and shifts macrophage phenotype. *FASEB Journal*.

[20] Chatterjee A., Sharma A., Chen M., Toy R., Mottola G., Conte M. S. (2014). The pro-resolving lipid mediator maresin 1 (MaR1) attenuates inflammatory signaling pathways in vascular smooth muscle and endothelial cells. *PLoS ONE*.

[21] Wang Y., Li R., Chen L. (2015). Maresin 1 inhibits epithelial-to-mesenchymal transition *in vitro* and attenuates bleomycin induced lung fibrosis *in vivo*. *Shock*.

[22] Nordgren T. M., Heires A. J., Wyatt T. A. (2013). Maresin-1 reduces the pro-inflammatory response of bronchial epithelial cells to organic dust. *Respiratory Research*.

[23] Serhan C. N., Dalli J., Karamnov S. (2012). Macrophage proresolving mediator maresin 1 stimulates tissue regeneration and controls pain. *FASEB Journal*.

[24] Sun X., Jiao X., Ma Y. (2016). Trimethylamine N-oxide induces inflammation and endothelial dysfunction in human umbilical vein endothelial cells via activating ROS-TXNIP-NLRP3 inflammasome. *Biochemical and Biophysical Research Communications*.

[25] Eftekhari A., Ahmadian E., Azarmi Y., Parvizpur A., Hamishehkar H., Eghbal M. A. (2016). *In vitro/vivo* studies towards mechanisms of risperidone-induced oxidative stress and the protective role of coenzyme Q10 and N-acetylcysteine. *Toxicology Mechanisms and Methods*.

[26] Liu L., Gao C., Chen G. (2013). Notch signaling molecules activate TGF-*β* in rat mesangial cells under high glucose conditions. *Journal of Diabetes Research*.

[27] Gao C., Chen G., Liu L. (2013). Impact of high glucose and proteasome inhibitor MG132 on histone H2A and H2B ubiquitination in rat glomerular mesangial cells. *Journal of Diabetes Research*.

[28] Chen X., Wang D.-D., Wei T., He S.-M., Zhang G.-Y., Wei Q.-L. (2016). Effects of astragalosides from Radix Astragali on high glucose-induced proliferation and extracellular matrix accumulation in glomerular mesangial cells. *Experimental and Therapeutic Medicine*.

[29] Börgeson E., Godson C. (2012). Resolution of inflammation: therapeutic potential of pro-resolving lipids in type 2 diabetes mellitus and associated renal complications. *Frontiers in Immunology*.

[30] Serhan C. N., Yang R., Martinod K. (2009). Maresins: novel macrophage mediators with potent antiinflammatory and proresolving actions. *Journal of Experimental Medicine*.

[31] Sasaki K., Urabe D., Arai H., Arita M., Inoue M. (2011). Total synthesis and bioactivities of two proposed structures of maresin. *Chemistry - An Asian Journal*.

[32] Viola J., Lemnitzer P., Jansen Y. (2016). Resolving lipid mediators maresin 1 and resolvin D2 prevent atheroprogression in mice. *Circulation Research*.

[33] Gong J., Wu Z.-Y., Qi H. (2014). Maresin 1 mitigates LPS-induced acute lung injury in mice. *British Journal of Pharmacology*.

[34] Li R., Wang Y., Zhao E. (2016). Maresin 1, a proresolving lipid mediator, mitigates carbon tetrachloride-induced liver injury in mice. *Oxidative Medicine and Cellular Longevity*.

[35] Hong S., Lu Y., Tian H. (2014). Maresin-like lipid mediators are produced by leukocytes and platelets and rescue reparative function of diabetes-impaired macrophages. *Chemistry and Biology*.

[36] Volpe C., Anjos P., Nogueira-Machado J. (2016). Inflammasome as a new therapeutic target for diabetic complications. *Recent Patents on Endocrine, Metabolic & Immune Drug Discovery*.

[37] Schroder K., Tschopp J. (2010). The inflammasomes. *Cell*.

[38] Yang C.-S., Shin D.-M., Jo E.-K. (2012). The role of NLR-related protein 3 inflammasome in host defense and inflammatory diseases. *International Neurourology Journal*.

[39] Zhou R., Tardivel A., Thorens B., Choi I., Tschopp J. (2010). Thioredoxin-interacting protein links oxidative stress to inflammasome activation. *Nature Immunology*.

[40] Hutton H. L., Ooi J. D., Holdsworth S. R., Kitching A. R. (2016). The NLRP3 inflammasome in kidney disease and autoimmunity. *Nephrology*.

[41] Feng H., Gu J., Gou F. (2016). High glucose and lipopolysaccharide prime NLRP3 inflammasome via ROS/TXNIP pathway in mesangial cells. *Journal of Diabetes Research*.

[42] Schnaper H. W., Hayashida T., Hubchak S. C., Poncelet A.-C. (2003). TGF-*β* signal transduction and mesangial cell fibrogenesis. *American Journal of Physiology - Renal Physiology*.

